# Stroke Seasonality and Weather Association in a Middle East Country: A Single Tertiary Center Experience

**DOI:** 10.3389/fneur.2021.707420

**Published:** 2021-10-18

**Authors:** Saeed A. M. Alghamdi, Mohammed A. Aldriweesh, Bayan A. Al Bdah, Muath A. Alhasson, Sultan A. Alsaif, Waleed A. Alluhidan, Faisal M. Almutairi, Mohammed A. Alskaini, Naser Alotaibi, Ali M. Al Khathaami

**Affiliations:** ^1^Division of Neurology, Department of Medicine, King Abdulaziz Medical City, National Guard Health Affairs, Jeddah, Saudi Arabia; ^2^College of Medicine, King Saud Bin Abdulaziz University for Health Sciences, Jeddah, Saudi Arabia; ^3^King Abdullah International Medical Research Center, Jeddah, Saudi Arabia; ^4^College of Medicine, King Saud Bin Abdulaziz University for Health Sciences, Riyadh, Saudi Arabia; ^5^King Abdullah International Medical Research Center, Riyadh, Saudi Arabia; ^6^Unaizah College of Medicine, Qassim University, Qassim, Saudi Arabia; ^7^College of Medicine, Almaarefa University, Riyadh, Saudi Arabia; ^8^College of Medicine, Imam Mohammad Ibn Saud Islamic University, Riyadh, Saudi Arabia; ^9^Department of Neurology, Prince Sultan Military Medical City, Riyadh, Saudi Arabia; ^10^Division of Neurology, Department of Medicine, King Abdulaziz Medical City, National Guard Health Affairs, Riyadh, Saudi Arabia

**Keywords:** ischemic stroke, hemorrhagic stroke, weather, risk factors, Saudi Arabia

## Abstract

**Background:** Stroke is a medical condition that leads to major disability and mortality worldwide. Some evidence suggests that weather and seasonal variations could have an impact on stroke incidence and outcome. However, the current evidence is inconclusive. Therefore, this study examines the seasonal variations and meteorological influences on stroke incidence and outcome in the largest city in Saudi Arabia.

**Methods:** From February 2016 to July 2019, we retrospectively reviewed data from all patients with acute ischemic (AIS) or hemorrhagic stroke (HS) admitted to the stroke unit in a tertiary academic center in Saudi Arabia. The corresponding daily meteorological data were obtained for the same period. We considered the months from November to March as the cold season and April to October as the hot season.

**Results:** The final cohort included 1,271 stroke patients; 60.89% (*n* = 774) cases occurred in the hot season, while 39.1% (*n* = 497) in the cold season. Males accounted for 69.6% (*n* = 884) of the cases. The proportion of ischemic stroke was 83.2% [hot season 83.9% (*n* = 649) vs. cold season 82.3% (*n* = 409)]. We found no statistically significant difference between seasons (hot or cold) in stroke incidence, severity [National Institutes of Health Stroke Scale (NIHSS)], hospital course (pneumonia, thromboembolism, intensive care stay, or length of stay), or outcome [modified Rankin scale (mRS) on discharge and death].

**Conclusions:** In Riyadh, Saudi Arabia, our study found no impact of weather or seasonal variations on stroke incidence, hospital course, or outcomes. However, our findings warrant further research in different country regions.

## Introduction

Stroke accounts for over 13 million cases and 5.5 million deaths annually (1). Globally, the overall age-standardized stroke incidence rates, deaths, disability-adjusted life years (DALYs) have decreased (1). However, absolute stroke numbers are increasing significantly in developing countries, like the Kingdom of Saudi Arabia (KSA) (1, 2). In KSA, stroke is highly prevalent, with an estimated 26,000 cases in 2016 (1). Moreover, an epidemiological model predicted an increase of 67% in the first stroke in the upcoming 10 years (3). The expected increase in stroke incidence shows how stroke directly impacts people's lives and the nation's economic prospects. Therefore, understanding the modifiable factors that influence stroke incidence is vital to understand and, if possible, formulate preventive measures.

Several modifiable risk factors are known to influence the stroke onset, such as hypertension (HTN) and diabetes mellitus (DM); however, a large and growing body of literature has investigated the relationship between environmental factors such as weather, meteorological factors, seasonal variations, air pollution, and stroke incidence (4–8).

Researchers were unable to produce consistent results if the weather can directly impact a person's health condition. However, some studies showed a link between weather and stroke risk (4–6, 9–16). On the other hand, different observational studies and a meta-analysis showed the opposite (7, 9, 16, 17). Data are scarce in the Arabian Gulf Cooperation Council (GCC) countries; however, Salam et al. reported a significant relationship between seasonal variation and stroke incidence in Qatar (4).

To the best of our knowledge, no similar studies about seasonality and stroke incidence have been done in KSA. Hereby, we study the seasonal variations and meteorological influences on stroke incidence by measuring the minimum, average, and maximum temperatures the day before the index event. We also investigate if atmospheric pressure, wind speed, and relative humidity in the hot and cold seasons impact stroke incidence.

## Materials and Methods

### Study Setting and Geographic Area

The study was conducted at King Abdulaziz Medical City, Riyadh, Saudi Arabia (KAMC-RYD) between February 2016 and July 2019. Riyadh is the capital city of Saudi Arabia (24.7136°N, 46.6753°E). It is located in the center of the country and the Arabian Peninsula, with more than 8 million inhabitants (18). The city has a hot desert climate with a long extreme dry and hot summer and short cool winter. Therefore, we considered the months from November 1 to March 31 as the cold season and from April 1 to October 31 as the hot season.

### Study Population

We screened all patients with ischemic stroke (IS) or intracerebral hemorrhage (ICH) who were admitted to the stroke unit. For this study, we included all patients who fulfilled the following criteria: (1) sudden neurological deficit resembling stroke within 24 h before arrival to the emergency department and (2) evidence of acute brain infarct or hemorrhage detected by brain computed tomography (CT) or magnetic resonance imaging (MRI) corresponding to the neurological deficit. A stroke neurologist confirmed the final diagnosis. We excluded all patients with transient ischemic attack (TIA), traumatic brain hemorrhage, and patients older than 80 years age who were bedridden, had a prior disabling stroke, had dementia, or had terminal cancer, as they are ineligible to admission to the stroke unit.

### Data Collection

We reviewed all electronic medical records for patients who fit the inclusion criteria for the following variables: date of admission, demographic data, vascular risk factors, treatment with reperfusion therapy [tissue plasminogen activator (tPA)] or endovascular treatment (EVT), modified Rankin scale (mRS) on admission, National Institutes of Health Stroke Scale (NIHSS) on admission and discharge, length of stay (LOS), and in-hospital complications and death. NIHSS, mRS, and in-hospital complications were collected at presentation and during the hospital stay by the treating team.

### Meteorological Data

Daily minimum/maximum/mean temperature (°C), atmospheric pressure (hPa), wind speed (m/s), and relative humidity (%) data were obtained from the Presidency of Meteorology and Environment (PME) in KSA between January 1, 2016, and December 31, 2019. In the analysis, we used the mean value of the meteorological variable on the month of the incident stroke.

### Statistical Analysis

Data analysis was conducted using STATA version 14.0 for Windows. Continuous variables were presented as means ± standard deviation or median (interquartile range). The categorical variables were presented as counts and percentages. A chi-square test was applied to examine the differences between frequencies. *t*-test, ANOVA, and Kruskal–Wallis test were used for testing continuous variables. Multivariate logistic regression was applied for predicting stroke presentation and outcomes after adjusting for weather and clinical characteristics of the patients. Predictor variables were included in the model based on the literature review and the bivariate analysis. Differences were considered statistically significant if *p*-value < 0.05 with two-sided testing.

### Ethical Approval

The study received approval from the Institutional Review Board (IRB) committee (RC19/384/R) at King Abdullah International Medical Research Center (KAIMRC), the Ministry of National Guard Health Affairs.

## Results

The final cohort included 1,271 stroke patients between February 2016 and July 2019. The demographic and clinical characteristics are presented in Table 1. Among all the cases, 60.89% (*n* = 774) strokes occurred in the hot season, while 39.1% (*n* = 497) in the cold season. Male gender accounted for 69.6% (*n* = 884), with no statistically significant difference in gender between seasons. Regarding the risk factors, HTN was the commonest at 73.4% (*n* = 933), followed by diabetes at 62.6% (*n* = 796) and dyslipidemia at 28.3% (*n* = 360), with no statistically significant difference in risk factors between seasons. The median NIHSS score at admission was 3 (0–42) in the hot season and 4 (0–42) in the cold season, with no statistically significant difference. The incidence of IS was 83.2% in the hot season (*n* = 649) vs. 82.3% in the cold season (*n* = 409), while hemorrhagic stroke accounted for 16.8% in the hot season (*n* = 125) vs. 17.7% in the cold season (*n* = 88). Among all cases, the commonest presenting neurological symptom was a motor weakness at 69% (*n* = 877), with no statistically significant difference between seasons. Speech and/or language complaint accounted for 45.9% (*n* = 584) of all cases, with statistically significant difference found between the two seasons [hot 38.8% (*n* = 301); cold 56.9% (*n* = 283); *p* ≤ 0.001]. Regarding meteorological factors, the mean temperature was 32.73 ± 4.28°C in the hot season and 18.27 ± 3.09°C in the cold season. Moreover, the mean atmospheric pressure was 1017.58 ± 2.78 hPa in the hot season and 1005.41 ± 4.91 hPa in the cold season. The mean wind speed was 4.94 ± 0.967 m/s in the hot season and 5.83 ± 1.24 m/s in the cold season, and the relative humidity was 37.41 ± 9.81% in the hot season and 16.04 ± 7.89% in the cold season. The laboratory findings in our cohort showed a mean hematocrit (0.436 ± 0.059 hct%), mean sodium (136.4 ± 3.8 mmol/L), mean blood urea nitrogen (BUN) (6.4 ± 3.6 mg/dl), and mean creatinine (93.9 ± 65.0 μmol/L), with no statistically significant difference found between the laboratory findings in both seasons. Among all, thrombolysis was given in 8.2% (*n* = 104); and 3.5% (*n* = 44) were treated with EVT.

**Table 1:** The demographic and clinical characteristics of patients with ischemic and hemorrhagic stroke in hot and cold seasons.

**Variable**	**All stroke** **(*N* = 1,271)** ***N* (%)**	**Hot season** **(*N* = 774)** ***N* (%)**	**Cold season** **(*N* = 497)** ***N* (%)**	***P***
Mean age (years) (SD)	60 ± 12	60 ± 12	61 ± 13	0.596
**Gender**				
- Male	884 (69.6)	546 (70.5)	338 (68)	0.338
- Female	387 (30.4)	228 (29.5)	159 (32)	
**Medical history**				
- Ischemic heart disease	132 (10.4)	85 (11)	47 (9.5)	0.384
- Hypertension	933 (73.4)	569 (73.5)	364 (73.2)	0.914
- Diabetes mellitus	796 (62.6)	475 (61.4)	321 (64.6)	0.247
- Dyslipidemia	360 (28.3)	220 (28.4)	140 (28.2)	0.922
- Atrial fibrillation on admission	85 (6.7)	43 (5.6)	42 (8.5)	0.044
- Smoking/history of smoking	204 (16.1)	111 (14.3)	93 (18.7)	0.038
Mean BMI (kg/m^2^) (SD)	29.05 ± 5.88	29.09 ± 6.03	28.99 ± 5.64	0.626
Median NIHSS score at admission (0–42) (IQR)	3 (0–100)	3 (0–65)	4 (0–100)	0.049
Mean SBP on admission (SD)	155.5 ± 28.5	155.4 ± 28.4	155.6 ± 28.5	0.868
Mean DBP on admission (SD)	83.9 ± 26.7	83.7 ± 30.8	84.2 ± 18.4	0.239
Median mRS before stroke (0–6) (IQR)	0 (0–1)	0 (0–1)	0 (0–1)	0.405
**Stroke subtype**				
- Ischemic stroke	1,058 (83.2)	649 (83.9)	409 (82.3)	0.468
- hemorrhagic stroke	213 (16.8)	125 (16.1)	88 (17.7)	
**Sudden neurological symptoms**				
- Speech and language	584 (45.9)	301 (38.8)	283 (56.9)	***<0.001***
- Motor weakness	877 (69)	517 (66.8)	360 (72.4)	0.052
- Sensory symptoms	425 (41.2)	274 (35.4)	151 (30.4)	0.023
- Gait imbalance	403 (31.7)	259 (33.5)	144 (28.9)	0.035
**Meteorological factors**				
Mean temperature (°C) (SD)	26.73 ± 8.14	32.73 ± 4.28	18.27 ± 3.09	
Mean atmospheric pressure (hPa) (SD)	1,010.46 ± 7.34	1,017.58 ± 2.78	1,005.41 ± 4.91	
Mean wind speed (m/s) (SD)	5.46 ± 1.2	4.94 ± 0.967	5.83 ± 1.24	
Mean humidity (%) (SD)	24.9 ± 13.71	37.41 ± 9.81	16.04 ± 7.89	
**Laboratory findings**				
Mean hematocrit (Hct%) (SD)	0.436 ± 0.059	0.434 ± 0.06	0.438 ± 0.063	0.533
Mean sodium (mmol/L) (SD)	136.4 ± 3.8	136.6 ± 0.056	136.2 ± 3.7	0.063
Mean BUN (mg/dl) (SD)	6.4 ± 3.6	6.3 ± 3.4	6.7 ± 4.0	0.431
Mean creatinine (μmol/L) (SD)	93.9 ± 65.0	94.9 ± 70.7	92.5 ± 55.1	0.720
Treatment given				
Thrombolysis (tPA)	104 (8.2)	64 (8.3)	40 (8.0)	0.889
Endovascular thrombectomy	44 (3.5)	25 (3.2)	19 (3.8)	0.568

*The bold p-value of < 0.05 was considered statistically significant*.

*BMI, body mass index; NIHSS, National Institutes of Health Stroke Scale; SBP, systolic blood pressure; DBP, diastolic blood pressure; mRS, modified Rankin scale; IS, ischemic stroke; ICH, intracranial hemorrhage; tPA, tissue plasminogen activator; BUN, blood urea nitrogen*.

We examined the effect of hot or cold seasons on stroke outcome; see Table 2. The median NIHSS score on discharge was 3 (1–9) in the hot season and 4 (1–8) in the cold season. Dependency on discharge, which is defined as mRS = 3–5, happened in 29.7% (*n* = 230) in the hot season and 29.8% (*n* = 148) in the cold season. Admission to the intensive care unit (ICU) was needed for 10.9% (*n* = 84) in the hot season and 11.3% (*n* = 56) in the cold season. Median LOS was 6 (3–15) days in the hot season and 7 (4–15) days in the cold season. Regarding the in-hospital complications, pneumonia happened in 4.8% (*n* = 37) in the hot season and 7.2% (*n* = 36) in the cold season; urinary tract infection in 7.4% (*n* = 57) in the hot season and 7.6% (*n* = 38) in the cold season; thromboembolism in 2.1% (*n* = 16) in the hot season and 0.8% (*n* = 4) in the cold season; and death in 7% (*n* = 54) in the hot season and 7.4% (*n* = 37) in the cold season. None of these outcome variables showed any statistically significant difference between seasons.

**Table 2:** Effect of seasons (hot or cold) on stroke outcomes.

**Outcome**	**All stroke** ***N* (%)** **(*n* = 1,271)**	**Hot** ***N* (%)** **(*n* = 774)**	**Cold** ***N* (%)** **(*n* = 497)**	***P***
Median NIHSS score on discharge (0–42) (IQR)	3.0 (1.0–8.0)	3.0 (1.0–9.0)	4.0 (1.0–8.0)	0.816
Dependent on discharge^*^	378 (29.7)	230 (29.7)	148 (29.8)	0.981
ICU admission	140 (11.0)	84 (10.9)	56 (11.3)	0.795
Median LOS (days)	6.0 (3.0–15.0)	6.0 (3.0–15.0)	7.0 (4.0–15.0)	0.528
**Medical complications**				
- Pneumonia	73 (5.7)	37 (4.8)	36 (7.2)	0.066
- Urinary tract infection	95 (7.5)	57 (7.4)	38 (7.6)	0.852
- Deep vein thrombosis/pulmonary embolism	20 (1.6)	16 (2.1)	4 (0.8)	0.078
- Death	91 (7.2)	54 (7.0)	37 (7.4)	0.752

*A p-value of < 0.05 was considered statistically significant*.

*NIHSS, National Institutes of Health Stroke Scale; ICU, intensive care unit; LOS, length of stay; mRS, modified Rankin scale*.

* *Check only those with mRS (3–5) on discharge and compare among seasons*.

Table 3 shows a multivariate logistic regression model to examine the impact of a 1-unit increase in the temperature, relative humidity, wind speed, and atmospheric pressure on stroke outcomes. Adjusted odds ratios (AORs) with confidence interval were reported. One-unit increase in mean relative humidity resulted in statistically significant odds of change in NIHSS on discharge (mean relative humidity 21.3%: AOR 0.98; 95% CI: 0.96–0.99; *p* = 0.041). Also, a 1-unit increase in mean atmospheric pressure resulted in statistically significant odds of a different outcome in ICU admission (mean atmosp. 1009.5 hPa; AOR: 0.93; 95% CI: 0.87–0.99; *p* = 0.045) and death (mean atmosp. 1009.0 hPa; AOR: 0.9; 95% CI: 0.83–0.99; *p* = 0.022). Table 4 presents the incidence of stroke according to the month. Again, there was no significant difference in stroke incidence among all months of the year (*p* = 0.897).

**Table 3:** Multivariate regression analysis to examine the impact of 1-unit increase in the temperature, relative humidity, wind speed, and atmospheric pressure on stroke outcomes.

**Independent variable**	**Temp. (°C) (Mean)**	**Adjusted odds ratio (95% CI)** ***P***	**Relative humidity** **(%)** **(Mean)**	**Adjusted odds ratio (95% CI)** ***P***	**Wind speed** **(m/s)** **(Mean)**	**Adjusted odds ratio (95% CI)** ***P***	**Atmosp. pressure** **(hPa)** **(mean)**	**Adjusted odds ratio (95% CI)** ***p***
NIHSS score on discharge (>7)	27.1	0.94 (0.89–1.01) *p* = 0.091	21.3	0.98 (0.96–0.99) ***p** **=*** 0.041*	5.0	0.94 (0.87–1.015) *p* = 0.121	1,010.2	0.95 (0.89–1.01) *p* = 0.102
mRS on discharge (>2)	26.7	0.98 (0.93–1.03) *p* = 0.414	23.2	0.99 (0.98–1.01) *p* = 0.716	5.4	0.95 (0.89–1.01) *p* = 0.083	1,009.9	0.97 (0.92–1.02) *p* = 0.180
ICU admission	27.3	0.96 (0.89–1.03) *p* = 0.209	25.0	1.01 (0.99–1.02) *p* = 0.218	5.6	0.99 (0.91–1.07) *p* = 0.776	1,009.5	0.93 (0.87–0.99) ***p*** **=** **0**. 045*
LOS (>1 day)	26.8	0.98 (0.91–1.07) *p* = 0.716	24.3	1.01 (0.99–1.04) *p* = 0.229	5.5	1.02 (0.93–1.12) *p* = 0.668	1,010.3	1.01 (0.93–1.1) *p* = 0.827
**Medical complications**								
- Pneumonia	25.4	0.93 (0.84–1.02) *p* = 0.122	26.9	1.0 (0.98–1.02) *p* = 0.803	5.5	1.0 (0.91–1.12) *p* = 0.856	1,011.2	0.95 (0.86–1.04) *p* = 0.236
- UTI	26.7	1.0 (0.92–1.09) *p* = 0.912	25.0	1.0 (0.99–1.02) *p* = 0.671	5.5	1.01 (0.92–1.11) *p* = 0.879	1,010.5	1.0 (0.93-1.09) *p* = 0.877
- DVT/PE	31.8	0.89 (0.73–1.09) *p* = 0.262	16.4	0.98 (0.92–1.04) *p* = 0.518	6.0	0.98 (0.81–1.18) *p* = 0.812	1,004.8	0.83 (0.68–0.99) *p* = 0.05
- Death	27.7	0.935 (0.86–1.02) *p* = 0.129	23.7	1.0 (0.99–1.02) *p* = 0.614	5.4	0.95 (0.86–1.05) *p* = 0.278	1,009.0	0.90 (0.83–0.99) p = 0.022*

*The bold p-value of < 0.05 was considered statistically significant*.

*CI, confidence interval; NIHSS, National Institutes of Health Stroke Scale; mRS, modified Rankin scale; ICU, intensive care unit; LOS, length of stay; UTI, urinary tract infection; DVT, deep vein thrombosis; PE, pulmonary embolism*.

**Table 4:** The incidence of ischemic and hemorrhagic stroke in Riyadh, Saudi Arabia, by months between 2016 and 2019.

**Month**	**Incidence of stroke *N* (%)** **(*n* = 1,271)**	**Stroke classification**	
		**Ischemic *N* (%) (*n* = 1,058)**	**Hemorrhagic *N* (%) (*n* = 213)**
January	90 (7.1)	73 (6.9)	17 (8)
February	143 (11.3)	116 (11.0)	27 (12.7)
March	107 (8.4)	92 (8.7)	15 (7.0)
April	140 (11.0)	120 (11.3)	20 (9.4)
May	126 (9.9)	100 (9.5)	26 (12.2)
June	117 (9.2)	101 (9.5)	16 (7.5)
July	120 (9.4)	99 (9.4)	21 (9.9)
August	89 (7.0)	73 (6.9)	16 (7.5)
September	93 (7.3)	81 (7.7)	12 (5.6)
October	89 (7.0)	75 (7.1)	14 (6.6)
November	82 (6.5)	67 (6.3)	15 (7.0)
December	75 (5.9)	61 (5.8)	14 (6.6)

## Discussion

The present study was designed to examine the relationship between weather and stroke incidence and outcome. The study cohort had 1,271 patients with ischemic and hemorrhagic strokes. The majority of the cases occurred in the hot season (60.89%). Our data suggest but does not confirm that season does not affect stroke outcomes. We found a negative correlation between meteorological factors, including daily mean temperature, atmospheric pressure, and stroke incidence.

Nevertheless, we examined the impact of a 1-unit increase in all chosen weather variables after adjusting for demographic variables and potential risk factors. We found a statistically significant weather impact on stroke outcome with relative humidity on NIHSS and atmospheric pressure on ICU admission and death. There was a marginal significance and a trend of atmospheric pressure influence on thromboembolism. We cannot formulate a scientifically sound hypothesis to explain these findings. The intuitive idea that dehydration in the summer season and its impact on blood viscosity, electrolytes, and sodium levels might have contributed to these findings are not supported by significant differences between groups in our cohort in hematocrit or sodium levels. Therefore, we think that these statistical differences are merely driven by chance, poor generalizability with a single-center cohort, or a relatively small sample.

The discrepancy between seasonal variations and stroke incidence and outcome in many published papers makes it difficult to conclude the potential relationship. In our study, we found no such relationship. This is also the case in previously published papers (7, 9, 16, 17, 19–22). A large study with data extending up to 40 years, and more than 300,000 ischemic and hemorrhagic strokes, found neither seasonal nor meteorological association with stroke incidence (7). Moreover, a recent meta-analysis analyzed the data from 33 studies with more than 200,000 participants found unremarkable seasonal variation (17).

On the other hand, several studies linked the weather or seasonal variations with stroke incidence (4–6, 9, 10, 13–15, 23, 24). Locally, in Qatar, Salam et al. reported a significant relationship between seasonal variation and incidence of IS relative to ICH in summer (4). Nevertheless, a systematic review with 20 studies found that short-term ambient temperature changes are significantly associated with increased stroke incidence (25). Collectively, these studies outline inconsistent conclusions where most of them found an observational relationship only; this is possibly explained by different factors, including the number and characteristics of patients, geographic area, meteorological factors used, metabolic factors, and environmental factors. Hypothetically, dehydration, especially in summer, increases blood viscosity, making the risk of stroke greater (26, 27). However, applying this hypothesis to our sample through sodium, hematocrit, BUN, and creatinine found no statistically significant difference among seasons.

Some other environmental factors were investigated for a possible influence of stroke incidence and mortality, such as air pollution, particulate matter (PM), and carbon monoxide (CO) (8, 28). Surprisingly, Zhang et al. found PM to be positively correlated with IS only (8). The relationship might be explained at the cellular level, where air pollution and PM can induce inflammation, endothelial injury, atherosclerosis, and decreased cerebral blood velocity (29–32). Unfortunately, this was beyond the scope of our study and could not be examined in our sample.

Finally, our study has several important limitations. It is a retrospective cohort with the potential to miss some cases and data on several variables. We did not include air pollution and other toxicological factors such as CO. Also, this is a single-center study with a short time span that cannot be generalized to all KSA regions, as it has major variations in weather characteristics. In the data analysis, we choose dichotomous variables with their limitations for a reason. It is easier to interpret and analyze, and the daily variations in meteorological variables within a season in this part of KSA are minimal, as indicated in the standard deviations reported on all these variables.

## Conclusion

In Riyadh, Saudi Arabia, neither weather nor seasonal variations impact stroke incidence or outcome. Thus, our study might explain the cause–effect results in the previous observational studies, requiring an adjustment for the confounding factors.

## Data Availability Statement

The raw data supporting the conclusions of this article will be made available by the authors, without undue reservation.

## Author Contributions

SAlg and MAld wrote the introduction and results and helped with statistical analysis. BA did the statistical analysis. MAld, MAlh, SAls, WA, FA, and MAls collected the data, reviewed the literature, and co-wrote the results. NA and AA reviewed and edited the final manuscript. SAlg wrote the discussion and reviewed the literature. All authors contributed to the article and approved the submitted version.

## Conflict of Interest

The authors declare that the research was conducted in the absence of any commercial or financial relationships that could be construed as a potential conflict of interest.

## Publisher's Note

All claims expressed in this article are solely those of the authors and do not necessarily represent those of their affiliated organizations, or those of the publisher, the editors and the reviewers. Any product that may be evaluated in this article, or claim that may be made by its manufacturer, is not guaranteed or endorsed by the publisher.
